# Stroke Minimization through Additive Anti-atherosclerotic Agents in Routine Treatment (SMAART): study protocol for a randomized controlled trial

**DOI:** 10.1186/s13063-018-2564-0

**Published:** 2018-03-14

**Authors:** Fred Stephen Sarfo, Osei Sarfo-Kantanka, Sheila Adamu, Vida Obese, Jennifer Voeks, Raelle Tagge, Vipin Sethi, Bruce Ovbiagele

**Affiliations:** 1 Division of Neurology, Department of Medicine, Kwame Nkrumah University of Science and Technology, P.M. B, Kumasi, Ghana; 2 Komfo Anokye Teaching Hospital, Kumasi, Ghana; 3 Department of Neurology, Medical University of South Carolina, South Carolina, USA; 4 Cadila Pharmaceuticals, Ahmedabad, India

**Keywords:** Polypill, Secondary risk reduction, Stroke, Low- and middle-income countries, Carotid intima-media thickness

## Abstract

**Background:**

There is an unprecedented rise in the prevalence of stroke in sub-Saharan Africa (SSA). Secondary prevention guidelines recommend that antihypertensive, statin and antiplatelet therapy be initiated promptly after ischemic stroke and adhered to in a persistent fashion to achieve optimal vascular-risk reduction. However, these goals are seldom realized in routine clinical care settings in SSA due to logistical challenges.

We seek to assess whether a polypill containing fixed doses of three antihypertensive agents, a statin and antiplatelet therapy taken once daily per os for 12 months among recent stroke survivors would result in carotid intimal thickness regression compared with usual care (UC).

**Methods:**

The Stroke Minimization through Additive Anti-atherosclerotic Agents in Routine Treatment (SMAART) trial is a phase 2, open-label, evaluator-blinded trial involving 120 Ghanaian recent-ischemic-stroke survivors. Using a computer-generated sequence, patients will be randomly allocated 1:1 into either the intervention arm or UC. Patients in the intervention arm will receive Polycap DS® (containing aspirin, 100 mg; atenolol, 50 mg; ramipril, 5 mg; thiazide, 12.5 mg; simvastatin, 20 mg) taken as two capsules once daily. Patients in the UC will receive separate, individual secondary preventive medications prescribed at the physician’s discretion. Both groups will be followed for 12 months to assess changes in carotid intimal thickness regression – a surrogate marker of atherosclerosis – as primary outcome measure. Secondary outcome measures include adherence to therapy, safety and tolerability, health-related quality of life, patient satisfaction, functional status, depression and cognitive dysfunction.

**Discussion:**

An efficacy-suggesting SMAART trial could inform the future design of a multi-center, double-blinded, placebo-controlled, parallel-group, randomized controlled trial comparing the clinical efficacy of the polypill strategy for vascular risk moderation among stroke survivors in SSA.

**Trial registration:**

ClinicalTrials.gov, ID: NCT03329599. Registered on 11 February 2017.

**Electronic supplementary material:**

The online version of this article (10.1186/s13063-018-2564-0) contains supplementary material, which is available to authorized users.

## Background

There has been an astronomical rise in the prevalence of stroke in sub-Saharan Africa (SSA) which, when compared to stroke profiles in high-income countries (HIC) is characterized by a younger age of onset, higher early and long-term fatality rates, and more severe cognitive deficits, emotional and social isolation and disability among survivors [1–10]. Stroke survivors in SSA (vs. HIC) are especially at high risk for recurrent vascular events or death due to several contextural factors including uncoordinated health systems, under-controlled vascular risk factors [11, 12], and lack of care affordability.

Among stroke survivors, a major source of subsequent mortality and functional decline is from recurrent stroke and myocardial infarction (MI) [13–17]. Prevention of future vascular events is critical to reducing the morbidity/mortality of patients with stroke, since the risk is highest within 1 year of the index stroke [18, 19]. Antihypertensive, antithrombotic, anticoagulant, antidiabetic and lipid-lowering therapies form the foundation of modern secondary preventive strategies for strokes and other types of cardiovascular disease (CVD) [20]. Combination therapy using a statin, aspirin and antihypertensive agents has been associated with reductions in stroke, myocardial infarction (MI) and mortality risk compared to monotherapy [21], and an 80% reduction in overall cardiovascular event risk when aspirin, beta-blockers, lipid-lowering drugs and angiotensin-converting enzyme inhibitors (ACEIs) are used simultaneously [22]. Hence, most guidelines recommend that secondary prevention interventions for stroke (comprising antihypertensive, statin and antiplatelet therapy) should be initiated promptly after stroke and adhered to in a persistent fashion to achieve the goals of risk reduction for vascular events [23, 24]. However, these goals are seldom realized in routine clinical care settings, especially in Africa, due to logistical and affordability challenges [25–35]. Of note, the neurologist:population ratio in SSA ranges from 1 per 162,885 persons to none in 11 countries (vs. 1 per 29,200 persons in the US) [36], underscoring the need to identify relatively simple strategies that can be applied broadly for stroke survivors in resource-constrained settings.

The use of evidence-based therapies for vascular risk reduction among stroke patients receiving conventional care in low- and middle-income countries (LMIC) is extremely low [37]. Usually, an individual recovering from stroke is prescribed multiple medications to treat various risk factors indefinitely, and this often engenders poor adherence and non-persistence with these efficacious, evidence-based, preventive therapies [25–35]. Indeed, reports emanating from HIC suggest that continual utilization of secondary prevention medications is challenged, with short-to-medium-term persistence rates ranging between 60 to 90% [29–34]. Thus, non-adherence, treatment complexity, pill burden and limited expert input are the principal antagonists militating against widespread compliance to life-saving cardiovascular-disease-prevention medication interventions, leading the World Health Organization (WHO) to advocate for interventions that will address the factors contributing to non-adherence to CVD treatments as a priority issue [38].

Fixed-dose combination pills, also known as “polypills,” containing generic drugs: aspirin, a statin and blood pressure (BP)-lowering medication(s) may be a viable low-cost avenue to broadly improve medication adherence and consequently reduce further disability or death on a large scale among stroke survivors in SSA [39–54]. The principal objectives for the polypill strategy are improving drug adherence by reducing pill burden and thereby improving risk factor control and potentially reducing vascular event risk as a cost-effective intervention [44]. Feasibility studies have been conducted using polypills vs. “usual care,” (UC) with strong evidence for improved adherence [55–57], superior, or at least non-inferior, efficacy in systolic BP (SBP) and low-density lipoprotein cholesterol (LDL-C) control [40, 41, 46, 50, 55–57], better acceptability [55–57] and comparable safety profile and better cost-effectiveness in both LMIC and HIC [58–61]. Although these benefits are yet to translate into significantly higher reductions in hard outcomes vs. UC, the majority of the available studies have had a relatively short duration of follow-up (on average 8 weeks to 6 months) whereas it has been previously established that there is at least a 12–24-month lag phase before the maximum benefits of SBP and LDL-C reduction by these medications are typically observed [62, 63].

Hence, the overarching objective of the Stroke Minimization through Additive Anti-atherosclerotic Agents in Routine Treatment (SMAART) trial is to assess whether a polypill containing fixed doses of three antihypertensive agents, a statin and antiplatelet therapy taken once daily per os for 12 months would result in carotid intimal thickness regression, improved adherence, and tolerability compared with the UC group on separate individual secondary preventive medications among Ghanaian first-time stroke survivors.

## Methods

### Trial design

**Fig. 1:**
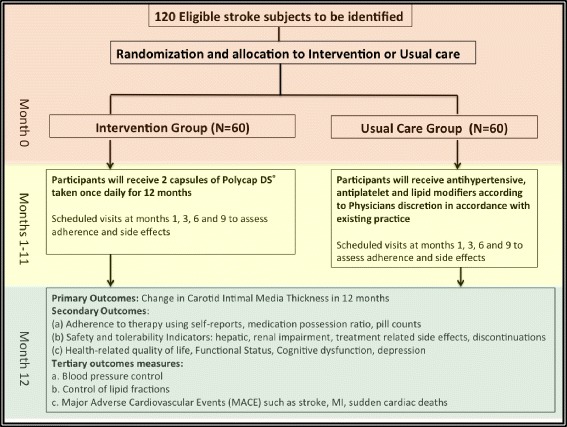
Stroke Minimization through Additive Anti-atherosclerotic Agents in Routine Treatment (SMAART) trial algorithm

SMAART is a phase II, randomized, open-label, blinded-endpoint clinical trial to evaluate the effect of a polypill taken once daily per os in improving CIMT regression and adherence to secondary risk reduction drug intake vs. a UC group with separate individual secondary preventive medications among Ghanaians with recent-onset stroke or transient ischemic attack (TIA) in a single-center study. See Fig. 1 for the study algorithm.

### Study settings

SMAART will be conducted at the Komfo Anokye Teaching Hospital (KATH), a tertiary-referral center and teaching hospital with 1000 beds. There is a stroke unit and a neurology outpatient service [64]. Stroke survivors encountered at the Family Medicine, Internal Medicine and Neurology Outpatient Clinics will be enrolled into the trial to capture UC experiences of stroke patients as well as the use of the polypill in these diverse settings. Each year, on average 450 stroke survivors are discharged from the hospital and approximately 300 stroke survivors are seen at the Family Medicine, Internal Medicine, Neurology Outpatient Clinics at KATH. Of note, the site has built up experience in stroke epidemiological studies as well as clinical trials involving stroke survivors [65–67].

### Study participants

The participants will include 120 adult Ghanaian recent stroke/TIA patients (within 2 months of stroke onset) meeting the inclusion/exclusion criteria who will be randomly assigned to either the intervention or the UC arm.

#### Inclusion criteria

1. Above the age of 18 years; male or female
2. Stroke/TIA diagnosis no longer than 2 months before enrollment. Only ischemic strokes including lacunar and large-vessel atherosclerotic subtypes are eligible
3. Legally competent to sign informed consent

#### Exclusion criteria

1. Unable to sign informed consent and having no proxy
2. Contraindications to any of the components of the polypill
3. Severe cognitive impairment/dementia or severe global disability limiting the capacity of self-care
4. Severe congestive cardiac failure (NYHA grades III–IV)
5. Severe renal disease, estimated glomerular filtration rate (eGFR) < 30 ml/min/1.73 m^2^), renal dialysis; awaiting renal transplant, or transplant recipient
6. Cancer diagnosis or treatment in the past 2 years
7. Nursing/pregnant mothers
8. Participants not agreeing to the filing, forwarding or use of their pseudonymized data

### Case definition

The diagnosis of stroke will be defined as an acute episode of focal cerebral, spinal or retinal dysfunction caused by infarction of central nervous system tissue, not resulting in death. Patients meeting the eligibility criteria will be allocated to the experimental or active comparator arm. Subjects with stroke may also present with at least one of the following additional conditions: documented diabetes mellitus or previous treatment with an orally administered hypoglycemic agent or insulin; documented hypertension (HT) > 140/90 mmHg or previous treatment with antihypertensive medications; mild to moderate renal dysfunction (eGFR 60–30 ml/min/1.73 m^2^); or prior MI.

### Intervention

Patients allocated to the experimental arm will receive two Polycap DS® (containing aspirin, 100 mg; atenolol, 50 mg; ramipril, 5 mg; thiazide, 12.5 mg; simvastatin, 20 mg) taken per os once a day. Polycap DS® is produced by Cadila Pharmaceuticals, Ahmedabad, India. Patients assigned to the polypill will have their antihypertensive agents, lipid modifiers and antithrombotic agents withdrawn and replaced with the polypill if they are already receiving such treatments before enrollment. Since our focus is to isolate the effect of the polypill strategy itself and create equipoise, at study inception providers for patients in both study arms will receive a brief one-off training and a one-off email synopsis on guideline-recommended biomarker targets after stroke [23]. To maximize participant’s adherence to the study medications, the following procedures will be implemented:

- Participant education during all visits on the importance of taking study medication, including timing, storage and what to do in the event of a missed dose
- Participants will be instructed to return unused capsules at each follow-up visit, and returned capsules will be counted and recorded by the study team
- Supervised dosing during face-to-face visits, when appropriate
- Application of the Morisky Green Questionnaire (MAQ) to participants at each yearly follow-up visit
- Participants will be provided with contact details of the responsible researcher so that they can make contact if for any reason they are unable to continue their study medication or have missed multiple doses and are unsure whether to continue

### Usual care group

Patients allocated to the UC arm will receive standard-of-care therapies for secondary prevention with drugs and doses left to the discretion of the treating physicians. Measures outlined to ensure adherence to study medications will be also be deployed in the UC group.

#### Management of possible treatment-related side effects

The following strategies will be employed for patients with side effects deemed due to study medication and sufficiently severe to warrant a change of treatment:

- If there are clear contraindications to one or more of the antihypertensive agents – ACEI, beta-blocker or thiazide; antithrombotic agent or lipid modifiers due to side effects, the polypill will be stopped and an open-label treatment with other components of the polypill excluding the class(es) for which the contraindication has developed
- If there are symptoms of severe hypotension, one approach will be to stop the polypill, introduce the individual components gradually at reduced doses and consider re-starting at a later date
- Indication for a high dose of a particular agent – the agent will be added as open label without the need to unblind

#### Discontinuation of study treatment

Participants who have had the study drug discontinued for any reason other than the above should be encouraged to restart the study drug as soon as practically and medically appropriate at the discretion of the investigator.

The investigator must not deviate from the protocol except when there is a contraindication due to the patient’s condition or the patient is intolerant of the drugs. In these cases the study treatment should be discontinued. Study treatment should also be discontinued if any of the following occurs:

1. Serious adverse events (SAEs) which are, in the opinion of the investigator, related to the study treatment
2. The investigator feels it is in the subject’s best interest

In the event of discontinuation of treatment, the patient should still remain in follow-up and attend study visits as scheduled. Physicians would then prescribe other medications for risk factor control and reasons for discontinuation documented.

#### Relevant concomitant care and interventions permitted or prohibited during the trial

Other medications deemed necessary for the management of other comorbidities, such as diabetes mellitus, will be permitted.

### Study procedures

#### Recruitment of study subjects

A total number of 120 patients will be randomized (1:1) to treatment arms. Patients with stroke meeting the inclusion/exclusion criteria will be recruited from the Family Medicine, Internal Medicine and Neurology Outpatient Clinics at KATH. Randomization will take place within 8 weeks of the index event (ischemic stroke) in a 1:1 ratio to one of the two arms: polypill versus UC.

#### Allocation and concealment

Randomization of subjects in blocks of 4 will be conducted by a statistician using a computer-generated random sequence of numbers. Consented participants will be assigned to either study arm at the baseline visit using the computer-generated randomization sequence. The randomization sequence uses a minimization algorithm to ensure balance of prognostic factors such as stroke severity, an average of three recorded baseline SBP measurements, and whether or not they are taking background BP-lowering agents, lipid modifiers and antithrombotic agents. Each sequence generated will be kept concealed in an envelope which will be opened by the research coordinator in the presence of the consenting study participant at enrollment.

#### Blinding

Physicians and sonographers who will be assessing primary outcomes and research assistants assessing feasibility and secondary outcomes will remain blinded as to patients’ group status throughout the study.

#### Screening evaluation

All potentially eligible participants referred to, or who contact the study team directly, will undergo screening to determine their eligibility using the pre-specified criteria.

#### Enrollment evaluation

Information on stroke type from a cranial computed tomography (CT) scan performed within 10 days of stroke symptom onset will be reviewed by the principal investigator (PI) (FSS); stroke subtype information where available will be sought to classify ischemic stroke using the TOAST classification [68] into cardio-embolic, large-vessel and lacunar ischemic stroke. Stroke severity and functional status at enrollment will be assessed using the modified NIHSS [69] and Modified Rankin Score [70], followed by a detailed assessment of vascular risk factors, namely HT, diabetes mellitus, dyslipidemia, cigarette smoking, cardiac diseases (atrial fibrillation, ischemic heart disease, cardiomyopathies), from history and physical examination. Blood samples for baseline assessments of renal and liver function tests, lipid profile and HbA1_C_ will be collected and contraindications for study medications assessed. All concomitant medications will be recorded in the Case Report Form.

### Follow-up and outcome evaluations

**Fig. 2:**
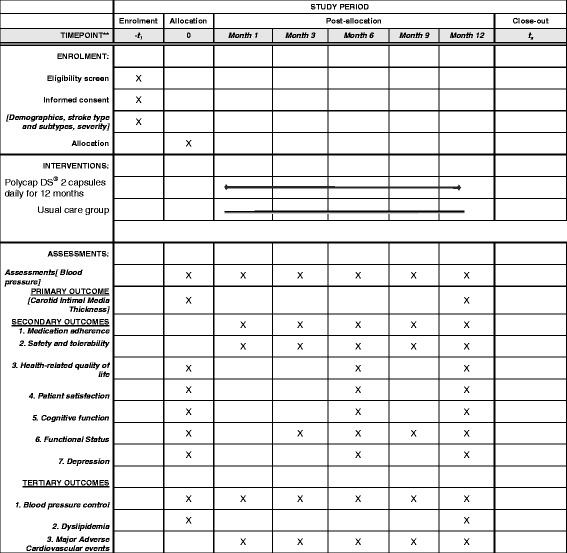
Standard Protocol Items: Recommendations for Interventional Trials (SPIRIT) Figure: SMAART trial protocol – schedule of enrollment, intervention and assessments

Participants will be followed for 12 months with scheduled visits at months 1, 3, 6, 9 and 12 for clinical assessments and primary, secondary and tertiary/feasibility study outcome evaluations as shown in Table 1 and the Additional file 1: Standard Protocol Items: Recommendations for Interventional Trials (SPIRIT) Checklist and SPIRIT Figure (Fig. 2). Prior to scheduled visits, patients will be reminded of their appointment 1 week and a day before the visit using telephone calls. Arrangements will be made for home visits for participants unable to attend visits or visits will be re-scheduled within 2 weeks where home visits are not feasible.

**Table 1:** Definitions of primary and secondary outcome measures to be assessed in the SMAART trial

Variable	Brief description
*Primary outcome measure* Carotid intima-media thickness (CIMT)	The end-of-study CIMT value will be subtracted from the baseline CIMT value and divided by the length of follow-up and the rate of change in CIMT (mm/year) between treatment arms and change in intima-media (artery wall) thickness and extent of atherosclerotic plaques in the carotid artery bifurcation measured
*Secondary outcome measures* Adherence to therapy	This will be measured at months 1, 3, 6, 9 and at month-12 clinic visits using the self-reported Morisky-Green Questionnaire (MAQ) [85], pill count and Medication Possession Ratio. Patients have to meet both criteria for adherence at the in-person visits to be considered adherent
Safety and tolerability indicators	Renal function: serum creatinine measurements to calculate eGFR using the CKD-EPI [86] formula at baseline and months 1 and 12 Liver function: elevations in liver enzymes will be assessed, if AST or ALT rises > ×5 Side effects’ profile: adverse events will be closely monitored and side effects will be documented according to the NIH/NCI Common Toxicity Criteria [87] at all scheduled and unscheduled study visitsDiscontinuation of medications: reasons and clinical indications for stopping treatment in both arms will be compared in both arms.Regimen adjustments: reasons for modifications in dosages including addition of new CVD agents, will be assessed in both treatment arms
Health-related quality of life	The EQ-5D questionnaire [88] will be used to assess state of health of study subjects at baseline and months 6 and 12
Change in patient satisfaction	The Treatment Satisfaction Questionnaire for Medication [89] will be administered at baseline and months 6 and 12
Cognitive dysfunction indicators	The Montreal Cognitive Assessment (MOCA) scale [90] will be used to assess global cognitive dysfunction at months 0, 6 and 12
Functional status	Functional status after stroke will be assessed using the modified Rankin Scale with a score from 0 to 6
Depression	Depression will be assessed using the Beck Depression Inventory and Hamilton Rating Scale for Depression at months 0, 6 and 12 [91, 92]
*Tertiary/feasibility* Cardiovascular risk factor control	1. BP control will be defined as SBP < 140 mmHg and/or DBP < 90 mmHg or (> 135/85 mmHg in diabetes patients). Mean change in SBP at month 12 from baseline will be compared in the two treatment groups 2. Dyslipidemia: control will be defined by change in mean LDL-C < 100 mg/dl or < 70 mg/dl. Mean change in LDL-C at month 12 from baseline will be compared in the two treatment groups
Incidence of adverse events	1. Recurrent stroke: fatal/severely disabling stroke or non-fatal stroke; coronary artery disease: acute STEMI/NSTEMI, sudden cardiac death 2. Re-hospitalization for any CVD cause; all-cause mortality

*BP* blood pressure, *DBP* diastolic blood pressure, *CVD* cardiovascular disease, *CKD-EPI* Chronic Kidney Disease Epidemiology Collaboration, *eGFR* estimated glomerular filtration rate, *EQ-5D* EuroQol five dimensions, *LDL-C* low-density lipoprotein cholesterol, *NIH/NCI* National Cancer Institute/National Institutes for Health, *NSTEMI* non-ST segment elevated myocardial infarction, *SBP* systolic blood pressure, *STEMI* ST segment elevated myocardial infarction

#### Primary outcome measure

The primary endpoint will be CIMT burden. Each study participant will undergo carotid Doppler ultrasonic evaluation at baseline and month 12 for evidence of clinical or sub-clinical carotid artery disease – a validated surrogate marker of atherosclerosis [71]. To achieve reliable ultrasonic measurements of the common carotid artery IMT a standardized protocol and strict quality control procedures will be followed by two local experienced sonographers who will be blinded to the participants group status and risk factor levels to ensure that we achieve unbiased results [72, 73]. CIMT will be measured at 1-cm portions of the distal left and right common carotid artery far walls with a linear transducer (transducer frequency of 7.5 MHz) with axial resolution of 0.10 mm, and calculated automatically over three cardiac cycles following the Mannheim consensus [73]. The average thickness of the left and right carotid arteries will be used as the outcome measure.

#### Secondary outcome measures

These will include (1) medication adherence indicators assessed using the self-reported Morisky-Green Questionnaire, pill count and medication possession ratio; (2) safety and tolerability indicators such as changes in renal and liver function tests, side effects and treatment discontinuations; (3) health-related quality of life; (4) patient satisfaction; (5) changes in cognitive function; (6) depression and (7) functional status.

#### Tertiary outcome measures

These will include CVD risk-factor-control indices, such as change in mean SBP between baseline and month 12, as well as changes in mean LDL-C from baseline to month 12. Major adverse cardiovascular events, such as recurrent stroke, MI, CVD-related deaths and all-cause mortality, are included as tertiary outcome measures.

#### Management of possible treatment-related side effects

Participants who experience side effects will be reviewed by their physicians to assess severity and appropriate measures instituted.

### Sample size justification

The end-of-study CIMT value will be subtracted from the baseline CIMT value and divided by the length of follow-up. The rate of change in CIMT (mm/year) between treatment arms will be tested with a two-sided *t* test. The rate of change in common CIMT in treated patients is around 0.085 mm/year with a standard deviation of 0.035 [74]. We assume that polypill improvement leads to a halting of CIMT progression with an assumed rate of change of 0.0825 mm/year. With a two-sided alpha of 0.05 and a 90% power we need 82 patients (104 patients with 20% dropout rate).

### Data management

All data entry will be collected onto a Case Record Form (CRF) and doubly entered into a password-protected, validated, encrypted study electronic CRF on REDCap. This web-based data management system will allow for real-time data query generation for values entered outside of pre-set valid ranges and consistency checking. This system will speed up data reporting and assist overall trial management. The Internet-based data management system is managed at the Kwame Nkrumah University of Science and Technology (KNUST) and hosted by the Medical University of South Carolina server, which has extensive experience in clinical trial data capture and security. Data entry will be performed at the KNUST/KATH using a password-protected, encrypted HTTPS connection. Only staff listed in the delegation log will be given the unique individual password to access the Internet-based data management system. The database will undergo logic checks to ensure that data are entered in mandatory fields and undergo value-range checks to ensure accuracy and to reduce the chance of missing data. Reports and data query management will also be included in the system to assist with centralized online monitoring by the data manager and statistician.

### Statistical analysis plan

Prior to addressing each hypothesis, univariate descriptive statistics and frequency distributions will be calculated as appropriate for all biological variables (including gender, age) comparing individuals by treatment arms (polypill arm vs. UC arm). Briefly, box plots will be used to examine the relative distribution of variables stratified by treatment arm. Non-parametric and equivalent parametric statistics will be utilized to compare groups. Appropriate regression models (linear regression for continuous outcomes such as carotid-media thickness; Cox proportional hazards regression for time-to-event measures such as recurrent strokes, CVD events, deaths and defaults) will be used to estimate the association of covariates with each outcome. When building models for each specific aim, the first stage of the model building algorithm will involve testing whether the individual covariates are correlated with the main outcome variables. A liberal alpha = 0.20 will be used for these unadjusted analyses [75]. Once the initial pool of candidate predictors has been identified, regression models consisting of multiple covariates will be fitted to identify potential confounders and effect modifiers. To achieve unbiased and robust results, optimal combinations of predictors, including interaction effects, will be identified and used for further analysis based upon whether or not they are a confounder, by whether they did not improve the model fit, or increased the standard error of the parameter estimate of the primary covariates. Finally, each model will be rigorously assessed for collinearity and goodness-of-fit using residual analysis. Model diagnostics will be performed using tools (in SAS or STATA) that detect outliers and influential data points. We will use diagnostic measures, such as residual deviance, the hat matrix diagonal and residual chi-squared deviance and the difference between chi-square goodness-of-fit, when an observation is deleted [76]. Plots of these against predicted values will be used to investigate the influence of each data point on the model. We will handle missing data using several techniques including multiple imputation and propensity score methods [77, 78].

### Data Safety Monitoring Board (DSMB)

A DSMB, comprised of three experienced external experts, will meet twice per year to review the safety, ethics and outcomes of the study. The responsibilities of the DSMB will include monitoring blinded response variables and safety outcomes for early dramatic benefits or potential harmful effects and provide reports on recommendations to continue or temporarily halt recruitment to the study. The DSMB will be governed by a charter that will outline their responsibilities, procedures and confidentiality, and will review unblinded data from the study at regular intervals during follow-up and monitor BP differences between the two groups, dropout rates and event rates. The first meeting will be held within 3–6 months after the start of the study recruitment. One or two formal interim analysis will be planned to review data relating to patient safety and quality of trial conduct.

### Harms

All SAEs and adverse events of special interest (AESI) experienced by a participant after the informed consent document is signed and until the end of the study will be collected and reported to the Institutional Review Board and applicable regulatory guidelines. If an SAE is unresolved at the conclusion of the study, a clinical assessment will be made by the medical monitor as to whether continued follow-up of the SAE is warranted.

## Discussion

The overarching objective of the proposed SMAART trial is to assess whether a polypill containing fixed doses of three antihypertensive agents, a statin and antiplatelet therapy taken once daily per os would result in carotid intimal thickness regression, improved adherence and tolerability among first-time stroke survivors in SSA. The ultimate objective would be the design of a future multi-center, double-blinded, placebo-controlled, parallel-group randomized controlled trial (RCT) comparing the clinical efficacy of the polypill strategy vs. UC in the African context to derive locally relevant, high-quality evidence for routine deployment of polypill for CVD risk moderation among stroke survivors in LMIC. There are six major RCTs on polypills currently on-going or planned, three of these RCTs – TIPS-3 [79], HOPE-3 [80] and HOPE-4 [81] – are primary prevention studies, two of them – PROPS [82] and SECURE [83] – are secondary prevention trials and PolyIran study [84] is aimed at both primary and secondary CVD prevention. As far as we are aware, a potential SMAART trial could be the first study to evaluate the feasibility of a polypill on secondary risk reduction among stroke survivors in SSA with the possibility of generating results that will inform the future research agenda and policy on the utility of the polypill for comprehensive vascular risk reduction in the region.

We anticipate the following potential challenges:

1. Subject accrual and retention: multiple tactics will be used to facilitate high patient retention including obtaining full contact information for patients, caregivers and relatives at time of enrollment and updating every 3 months. Transportation support will be provided when needed and follow-up visits will be scheduled, whenever possible, on days that subjects already have scheduled clinic visits
2. Meeting sample size: there is a high case load of stroke patients with approximately 700 cases per year. At a recruitment rate of five cases per week, recruitment of 120 stroke patients meeting eligibility criteria is a realistic objective
3. Missing data: we anticipate that there may be missing data due to loss to follow-up but we will minimize this by home visits where geographically feasible, providing transportation stipends and employing statistical methodology to account for missing data

### Trial status

Recruitment is anticipated to begin in April 2018.

## Additional file

Additional file 1: SPIRIT 2013 Checklist: recommended items to address in a clinical trial protocol and related documents. (DOC 121 kb)

## Abbreviations

ACEI: Angiotensin-converting enzyme inhibitor
ALT: Alanine transaminase
AST: Aspartate transaminase
BP: Blood pressure
CIMT: Carotid intima-media thickness
CT: Computerized tomography
CVD: Cardiovascular disease
DBP: Diastolic blood pressure
eGFR: Estimated glomerular filtration rate
EQ-5D: European Quality of Life Questionnaire-5 Dimensional
HIC: High-income countries
HOPE-3: Heart Outcomes Prevention Evaluation-3
HOPE-4: Heart Outcomes Prevention Evaluation-4
HTN: Hypertension
KATH: Komfo Anokye Teaching Hospital
LDL-C: Low-density lipoprotein cholesterol
LMIC: Low- and middle-income countries
MOCA: Montreal Cognitive Assessment
NIHSS: National Institute of Health Stroke Scale
NSTEMI: Non-ST segment elevated myocardial infarction
NYHA: New York Heart Association
PROPS: Preventative Role Of a fixed dose combination Pill in Stroke
RCT: Randomized controlled trial
SBP: Systolic blood pressure
SECURE: Secondary Prevention of Cardiovascular Disease in the Elderly Trial
SMAART: Stroke Minimization through Additive Anti-atherosclerotic Agents in Routine Treatment
SSA: Sub-Saharan Africa
STEMI: ST segment elevated myocardial infarction
TIPS-3: The International Polycap Study 3
UC: Usual care
WHO: World Health Organization
